# The Lock-Stitch in Surgery

**Published:** 1898-07-23

**Authors:** 


### THE LOCK-STITCH IN SURGERY.

Mr. Arthur Barker has devised an exceedingly ingenious arrangement, by which the form of stitch which is commonly used in what are called lock-stitch sewing machines is made available in surgical practice. He makes use of a newly-devised self-feeding needle, a needle holder containing a reel of thread or silk for suture, which can be paid out as required, and so fed into the needle. The latter can be held at any angle, and, having the eye near the point, can be made to perforate the tissues and leave a loop at the other side on withdrawal. The whole thing can be taken to pieces and boiled, and can, in fact, be boiled again after it is charged. The apparatus is used after this fashion. The needle being threaded is pushed through both the sides of the structures to be sutured. The free end of the suture is then pulled right through, and, being held, the needle is withdrawn. The needle then makes the second stitch through both sides, and on being slightly withdrawn a loop is at once formed on the far side. Through this loop the free end from the preceding stitch is passed, and the needle is then withdrawn, the finger pressing on the bobbin just enough to prevent the thread running loose. A third stitch is then made in the same manner, and so on all the way along, the thread which is in the needle always coming back to the needle side, and at each stitch pulling tight the loop which it has made on the other side—the loop through which the loose end of the thread has each time been tucked. Thus we have exactly the sort of stitch that is made by a lock-stitch sewing machine, a stitch in which a thread runs down each side of the fabric and the two interlock at each puncture. If the "tension" is kept right, this interlocking takes place in the thickness of the fabric, and so it does with Mr. Barker's lock-stitch. Some little practice is required, especially in regard to the tension. The advantages of the stitch are the absence of a row of knots and loose ends, as in the interrupted suture; the complete locking at each stitch, a thing which the continuous suture does not afford; and especially a great saving of time, a matter of prime importance in abdominal operations.

